# Grupo de Trabajo de la EFLM sobre Acreditación y Normas ISO/CEN sobre cómo abordar los requisitos de la norma ISO15189 sobre retención de documentación y muestras

**DOI:** 10.1515/almed-2024-0020

**Published:** 2024-03-08

**Authors:** Pika Meško Brguljan, Marc H.M. Thelen, Francisco A. Bernabeu-Andreu, Christos Kroupis, Guilaine Boursier, Ines Vukasović, Edward Barrett, Duilio Brugnoni, Maria Lohmander, Luděk Šprongl, Tatjana Vodnik, Irina Ghiţă, Florent Vanstapel, Michel Vaubourdolle, Willem Huisman

**Affiliations:** 68936 Hospital Universitario de Enfermedades Respiratorias y Alergias, Golnik, Eslovenia; Servicio de Análisis Clínicos, Hospital Universitario de Radboud, Nijmegen, Países Bajos; y Fundación de los Países Bajos para la Garantía de Calidad en los Análisis Clínicos (SKML), Nijmegen, Países Bajos; Servicio de Análisis Clinicos, 16370 H.U. Puerta de Hierro Majadahonda, Madrid, España; Servicio de Bioquímica Clínica, Hospital General Universitario Attikon, Facultad de Medicina, Universidad Nacional y Kapodistríaca de Atenas, Haidari, Grecia; Centro Hospitalario Universitario de Montpellier, Servicio de Genética, 26905 Enfermedades Raras y Medicina Personalizada, Montpellier, Francia; Centro Hospitalario Universitario Sestre Milosrdnice, Servicio de Análisis Clínicos, Zagreb, Croacia; Consultor de Bioquímica Clínica, Limerick, Irlanda; 224928 Laboratorio de Análisis Clínicos – Spedali Civili, Brescia, Italia; Servicio de Análisis Clínicos NU-Sjukvården, Trollhättan, Suecia; Laboratorio Clínico, 48388 Hospital Kladno, Kladno, República Checa; Centro de Bioquímica Clínica, Centro Clínico de Serbia, Belgrado, Serbia; Laboratorio Central de Ensayos Clínicos, condado de Iflov, Chiajna, Rumanía; Análisis Clinicos, Hospital Universitario de Leuven, Leuven, Belgica; y Servicio de Salud Pública, Grupo de Ciencias de la Biomedicina, KU Leuven, Leuven, Belgica; Hospital Saint-Antoine – AH-HP – Servicio de Bioquímica, Paris, France; Consultor especialista en química clínica y medicina de laboratorio, La Haya, Países Bajos

**Keywords:** EFLM, análisis modal de fallos y efectos (AMFE), ISO 15189, gestión de la calidad, tiempo de retención, evaluación de riesgos

## Abstract

Gran parte de la actividad en el laboratorio clínico debe quedar documentada para facilitar el mantenimiento de la calidad del servicio, lo que deriva en la generación de una gran cantidad de documentación, formularios e informes. Con tal objeto, es necesario especificar el tiempo de retención requerido para cada tipo de documento y muestra. Además de la obligación de conservar los informes del laboratorio clínico como parte de la historia clínica de los pacientes, el laboratorio también está obligado por ley, o según las directrices de las organizaciones profesionales, si las hubiera, a retener un gran volumen de documentación y muestras. En caso contrario, la dirección del laboratorio deberá establecer un calendario de retención, en el que se especifiquen las condiciones y el periodo de conservación, de acuerdo con los requisitos de la norma ISO 15189:2022 sobre retención de documentación y registros de gestión de la calidad.

En el presente proyecto, el Grupo de Trabajo de la EFLM sobre Acreditación y Normas ISO/CEN propone una serie de periodos de retención de documentación y muestras, basada en la metodología de análisis modal de fallos y efectos (AMFE), un método para la reducción de riesgos que ha pasado a formar parte integral de los estándares actuales.

## Introducción

Gran parte de la actividad en el laboratorio clínico debe quedar documentada para facilitar el mantenimiento de la calidad del servicio [1], [2], [3], [4], [5], lo que deriva en la generación de una gran cantidad de documentación, formularios e informes. El tiempo de retención requerido para cada tipo de documento debe ser especificado en el sistema de gestión de calidad del laboratorio [1], [2], [3].

Los informes del laboratorio clínico forman parte de la historia clínica de cada paciente, debiendo ser retenidos durante el tiempo establecido por la legislación de cada país. Dichos informes, también se pueden conservar en formato digital en el sistema de información del laboratorio o en papel durante un periodo de tiempo concreto, que puede llegar a superar los 100 años (toda la vida), en el caso de algunas pruebas concretas. Si no se han definido periodos concretos de retención, la dirección del laboratorio deberá definir un calendario de retención para los distintos tipos de documentos, formularios, registros y muestras. En dicho plan, se deberán definir las condiciones de almacenamiento (p.ej. en papel o en formato digital, en el caso de la documentación), así como el tiempo de conservación, según la norma ISO 15189:2022 [2].

De acuerdo con esta norma, la documentación se puede conservar en cualquier formato, siempre que se pueda acceder a la misma y se conserve de forma segura frente a modificaciones no autorizadas y deterioro injustificado. En las instalaciones se deberá contar con un emplazamiento adecuado para el almacenamiento de la documentación, con el fin de evitar daños, deterioro, pérdida o acceso no autorizado. En el caso de algunos documentos, especialmente aquellos conservados en formato electrónico, la forma más segura de almacenarlos será en medios seguros en una ubicación externa (o incluso en la nube, siempre y cuando se cuente con un nivel de seguridad adecuado). Aunque el periodo de retención de cada tipo de documento variará, los resultados de los pacientes deberán conservarse mientras sigan siendo clínicamente relevantes, o según lo establecido por ley. Así mismo, es necesario que los organismos de acreditación proporcionen directrices sobre los requisitos en materia de tiempo de retención.

El Grupo de trabajo de la Federación Europea de Química Clínica y Análisis Clínicos (EFLM) sobre Acreditación y Normas ISO/CEN publicó en 2011 un cuestionario destinado a evaluar las prácticas actuales en los países representados por las sociedades pertenecientes a la EFLM. El estudio reveló una gran heterogeneidad en las prácticas, lo que derivó en la elaboración de la presente propuesta. A continuación, exponemos las razones por las que consideramos idónea la metodología de análisis modal de fallos y efectos (AMFE) para establecer tiempos de retención para los distintos elementos del sistema de documentación del laboratorio. Los organismos reguladores ya habrán establecido tiempos de retención para los informes y las muestras de los pacientes.

## Materiales y métodos

### Cuestionario

En 2011, se envió a las sociedades pertenecientes a la EFLM un extenso cuestionario sobre todos los documentos enumerados en las cláusulas 4.13 y 5.7.2 de la norma ISO 15189:2012 [1], en las que se establecen los requisitos de retención de los informes de laboratorio y material diagnóstico.

### Análisis de riesgos

Originariamente desarrollados para la industria manufacturera, los principios de gestión de riesgos [6, 7] se aplican para la identificación y control de las fuentes de error analítico (CLSI EP18-A2, ISO/DIS 22367, CLSI EP23-A) [8], [9], [10]. Basándonos en el análisis de gestión de riesgos y aplicando la técnica AMFE, se pueden establecer tiempos de retención razonables, así como realizar una evaluación prospectiva de riesgos, para determinar si estos son realmente idóneos.

En la metodología AMFE, lo primero que se categoriza es la probabilidad de un riesgo (P):

**Table:** 

Probabilidad (P)	
Clasificación	Definición
A	Extremadamente improbable (prácticamente imposible o no se conoce ningún caso con productos o procesos similares, tras muchas horas de funcionamiento)
B	Remoto (relativamente pocos fallos)
C	Ocasional (fallos puntuales)
D	Razonablemente posible (fallos recurrentes)
E	Frecuente (el fallo es casi inevitable)

A continuación, se clasifica la gravedad (G):

**Table:** 

Gravedad (G)		
Clasificación	Definición
I	Sin efectos relevantes sobre la fiabilidad o la seguridad
II	Despreciable, no causaría daños ni perjuicios, implicaría únicamente una acción de mantenimiento (solo lo percibirían los clientes exigentes)
III	Muy pequeño, causaría un bajo nivel de daños, así como perjuicios leves (afecta a una pequeña parte del sistema, percibido por el cliente medio)
IV	Moderado, daño moderado, posibles perjuicios (la mayoría de los clientes están molestos, causa principalmente perjuicios económicos)
V	Crítico (causa una pérdida de su función principal; Pérdida de todos los márgenes de seguridad, a un fallo de provocar una catástrofe, daños graves, perjuicios graves, pudiendo causar incluso alguna muerte)
VI	Catastrófico (el producto deja de funcionar; el fallo puede provocar un funcionamiento muy peligroso, así como múltiples muertes)

A continuación, se combinan la probabilidad y la gravedad para clasificar el riesgo:

Finalmente, se evalúa la tasa de detección. En el caso de los tiempos de retención, existe la posibilidad de que se reconozca la necesidad de consultar la documentación o de repetir el análisis de la muestra antes de que esta sea eliminada.

**Table:** 

Clasificación de la detección	Definición
1	Segura – el fallo será detectado inmediatamente
2	Casi segura
3	Alta
4	Moderada
5	Baja
6	El fallo no es detectado por el usuario ni por el personal de mantenimiento

Cuanto menor sea la probabilidad de una detección a tiempo (mayor la clasificación en una escala del 1 al 6), menor será la categoría de riesgo que se puede considerar aceptable.

**Table:** 

Grado de riesgo (P×G)						
Probabilidad	Gravedad					
	I	II	III	IV	V	VI
A	Baja	Baja	Baja	Baja	Moderada	Alta
B	Baja	Baja	Baja	Moderada	Alta	Inaceptable
C	Baja	Baja	Moderada	Moderada	Alta	Inaceptable
D	Baja	Moderada	Moderada	Alta	Inaceptable	Inaceptable
E	Moderada	Moderada	Alta	Inaceptable	Inaceptable	Inaceptable

## Resultados y discusión

De acuerdo con las respuestas aportadas por nueve sociedades nacionales pertenecientes a la EFLM (tasa de respuesta 29 %), se observó una amplia dispersión en los tiempos de retención que se establecen en las cláusulas 4.13 y 5.7.2 de la norma ISO 15189:2012 [1] sobre documentación general de gestión de calidad, registros de personal, registros de sistemas, resultados analíticos y datos sin procesar, tal como expuso W. Huisman en la reunión de la EA (Acreditación Europea) de 2011, en su presentación (*“Results of EFLM WG ISO/CEN Accreditation survey on Requirements for the retention of laboratory records and diagnostic material”,* – comunicación individual) (Tabla Suplementaria 1) [11]. En la versión más reciente de la norma ISO 15189:2022, los tiempos de retención se establecen en las cláusulas 8.4.3 y 7.4.2 [2]. En comparación con la antigua norma ISO 15189:2012, la nueva versión, ISO 15189:2022, es menos prescriptiva con respecto a la documentación requerida, incluyendo los aspectos relacionados con los tiempos de retención. De este modo, según la nueva norma, es el laboratorio el que tiene que establecer los tiempos de retención. Aparte de los requisitos, también se pueden establecer los tiempos de retención, en función de los riesgos identificados [2].

El bajo índice de respuesta puede ser indicativo de la dificultad a la hora de realizar un inventario de prácticas en multitud de aspectos, lo que se ve reflejado en la diversidad de respuestas. En Alemania, existen requisitos legales específicos relativos a los datos genéticos, basados principalmente en los conceptos de privacidad, soberanía, esto es, derecho a no saber, y el concepto de excepcionalidad genética.

La necesidad de armonizar los tiempos de retención es evidente, razón por la cual el EFLM WG A/ISO ha elaborado la presente propuesta.

### Necesidades de los órganos de acreditación

Tal como expuso W.Huisman en la reunión de la EA (Acreditación Europea) de 2011, en su presentación (“*Results of EFLM WG ISO/CEN Accreditation survey on Requirements for the retention of laboratory records and diagnostic material*”, – comunicación individual) [11], [4, 5, 12], en multitud de países, el tiempo de retención se suele expresar como un periodo fijo de tiempo desde el momento en el que se creó el registro (p.ej. cuando se recibe una muestra), aunque también puede corresponder a un periodo de tiempo tras un evento concreto (p.ej. tras finalizar el estudio, tras el cese de la relación laboral en el caso de un registro de personal, etc.).

Dado que resulta difícil decidir qué tiempo de retención resulta razonablemente útil [12], las agencias reguladoras y de consultoría tienden a establecer tiempos de retención prolongados, con el fin de evitar la eliminación precipitada de documentación o material. Sin embargo, la retención ilimitada de documentos entra en conflicto con una gestión eficiente y un uso razonable de los recursos.

Una de las estrategias empleadas para racionalizar el tiempo de retención estándar para documentos de control de calidad o documentos de trazabilidad que permitan la realización de auditorías por parte de las agencias reguladoras, es aplicar el intervalo de tiempo entre dos auditorías, tal como sugieren algunos organismos de acreditación, como el COFRAC, el Comité Francés de Acreditación, que recomienda al menos veinticuatro meses por defecto [13, 14]. Según la norma ISO-15189:2022, es responsabilidad del laboratorio establecer los tiempos de retención. Aparte de los requisitos, también se pueden establecer los tiempos de retención en función de los riesgos identificados. Debido a algunos aspectos relacionados con la responsabilidad legal en algunos tipos de procedimientos (p.ej. análisis histopatológicos, estudios genéticos, exámenes en pacientes pediátricos), es posible que algunos informes requieran un periodo de retención más prolongado que otros [2].

### Análisis modal de fallos y efectos

Una estrategia es tratar de establecer una serie de directrices razonables que den una orientación sobre tiempos de retención útiles. A la luz de los rápidos avances experimentados en los últimos años en el ámbito de los análisis clínicos, así como a la implementación del concepto de reducción de riesgos, proponemos emplear un enfoque basado en el concepto de gestión de riesgos, con el fin de establecer tiempos de retención suficientemente largos como para evitar el riesgo de “*lamentar la eliminación*”, pero suficientemente cortos como para que sean prácticos y responsables en la utilización de recursos.

Con el fin de establecer tiempos de retención útiles, es necesario tener en cuenta, en primer lugar, las razones por las que pueden hacer falta los documentos y materiales, una vez ya han sido empleados para su uso original. Cuando coexisten varias razones para su retención, será la razón que requiera el mayor tiempo de retención la que determine el tiempo mínimo de retención.

### A continuación, se enumeran algunos ejemplos de razones para la retención de documentos relacionados

#### Revisión de la petición original

(a) Consulta iniciada por el laboratorio tras la autorización o comunicación de los resultados.
(b) Consulta iniciada por el médico solicitante tras utilizar los resultados del laboratorio recibidos.
(c) Reclamación por parte del médico solicitante tras utilizar los resultados del laboratorio.

Partiendo de estas posibles razones para retener la documentación y los materiales, se seleccionará la razón con una vigencia más prolongada para determinar el tiempo de retención. En dicho caso, esta es la razón (b). La razón (a) es la que tiene la vigencia más corta; una vez que el informe es enviado por el laboratorio, únicamente las razones expuestas por el médico solicitante podrán derivar en una revisión de la petición. Las razones (a) y (b) suelen coincidir. Desde el punto de vista del médico solicitante, las reclamaciones suelen surgir en el primer momento de uso de los resultados de la petición. Sin embargo, posteriormente se puede iniciar una consulta, por ejemplo, cuando el médico solicitante se reúna en la consulta con el paciente para informarle de los resultados. De este modo, un tiempo de retención razonable para la petición original podría ser el tiempo máximo transcurrido entre la petición y la siguiente cita de seguimiento del paciente.

Suponiendo que dicho periodo de tiempo no superará las ocho semanas, se puede realizar un análisis de riesgos AMFE para establecer el riesgo y evaluar si dicho periodo es demasiado corto.

**Table:** 

AMFE	
Tipo de error	Eliminar la petición original a las ocho semanas de haber enviado los resultados, pero antes de que el médico solicitante se haya puesto en contacto con el laboratorio para realizar alguna consulta sobre los resultados a raíz de la petición original
Probabilidad (P)	B (remota)
	En la mayoría de los casos, el médico tarda menos de ocho semanas en ver al paciente, desde que solicitó la prueba
Gravedad (G)	II (muy menor)
	Incluso cuando no se dispone de la petición original, se pueden realizar consultas sobre los resultados
Grado de riesgo (P×G)	Baja
Detección	6 (no detectado)
	El laboratorio no puede evitar que se elimine la petición si se tarda más de ocho semanas en ver al paciente desde que se solicitó la prueba
Acceptabilidad	Muy aceptable

Dado que se pueden realizar consultas independientemente de la disponibilidad o no de la petición, también se puede realizar el análisis AMFE de las probabilidades de que se elimine la petición antes de que el médico solicitante haya presentado una reclamación sobre el servicio de laboratorio sobre la petición (razón C).

**Table:** 

Tipo de error	Eliminar la petición original a las ocho semanas de haber enviado los resultados, pero antes de que el médico solicitante se haya puesto en contacto con el laboratorio para realizar alguna consulta sobre los resultados a raíz de la petición original
Probabilidad (P)	B (remota)
	En la mayoría de los casos, el informe se lee antes de que transcurran ocho semanas y, en la mayoría de los casos, no suele haber reclamaciones
Gravedad (G)	III (menor)
	Las controversias sobre el contenido de una petición, cuando está ya ha sido eliminada, pueden resultar tediosas, pero en ningún caso representarán ningún peligro
Grado de riesgo (P×G)	Baja
Detección	6 (no detectado)
	El laboratorio no puede evitar que se elimine el informe si el médico solicitante tarda más de ocho semanas en leerlo desde que este fue remitido
Acceptabilidad	Muy aceptable

#### Revisión de los resultados por parte del personal del laboratorio

(a) Comprobación de plausibilidad por parte del laboratorio antes de comunicar los resultados
(b) Comprobación de plausibilidad de los resultados por parte del laboratorio tras comunicarlos.

Dado que la comunicación de un nuevo resultado puede suscitar dudas sobre la precisión de otro resultado anterior, resulta razonable retener todos los datos originales necesarios para poder reconstruir un resultado, al menos hasta que se comunique un nuevo resultado de esa misma prueba. Esto implica, por ejemplo, que en el caso de una prueba X, después de un año, en la mayoría de los casos se habrá realizado un seguimiento y se dispondrá de algún resultado posterior. Los únicos resultados que se guardan por tiempo indefinido son aquellos que no suelen cambiar con el tiempo y para los que no se suele realizar una segunda prueba de seguimiento (pruebas genéticas de ADN).

Datos que es preciso conservar para poder reconstruir el resultado comunicado:

– Números de lote del reactivo
– Insertos de reactivos
– Datos de calibración
– Datos sobre los resultados sin procesar
– Datos de control de calidad
– Identidad del personal técnico implicado
– Cualificación de todo el personal implicado

**Table:** 

AMFE	
Tipo de error	Eliminar los datos originales necesarios para reconstruir el resultado original antes de disponer de un resultado más reciente, aunque haya transcurrido un año
Probabilidad (P)	B (remota)
	En la mayoría de los casos, se puede analizar la plausibilidad de un resultado, antes de disponer de otro más reciente. En la mayoría de los casos, un resultado reciente no será válido a la hora de evaluar la plausibilidad de un resultado anterior, si este último tiene más de un año de antigüedad
Gravedad (G)	III (menor).
	Cuando es poco probable que se vaya a repetir la prueba pero, transcurrido un año, resulta poco razonable cuestionar la validez de los resultados
Grado de riesgo (P×G)	Baja
Detección	4 (moderado)
	El laboratorio puede establecer un periodo de retención específico para las pruebas para las que pueda resultar útil un periodo de retención mayor, como es el caso de los resultados de las pruebas de ADN
Acceptabilidad	Muy aceptable

De este modo, toda la documentación, solicitudes y registros podrán ser analizados. Una forma eficaz es establecer escalas temporales para los registros de control interno de calidad, la gestión de reclamaciones y otros aspectos relacionados; por ejemplo, ocho semanas para los datos sin procesar de los instrumentos, los datos sobre las personas que recibieron la muestra y la manipularon en el laboratorio, o el tiempo transcurrido entre las visitas de control de evaluación por parte de los organismos de acreditación (en la mayoría de los países, un año). Para los registros de control externo de calidad, se puede establecer un plazo que coincida con el periodo de tiempo entre las evaluaciones de reacreditación (entre cuatro y cinco años, en la mayoría de los países).

### Reción de muestras y especímenes

El laboratorio deberá establecer el tiempo de retención de las muestras clínicas. Los tiempos de retención dependen de la naturaleza de la muestra, su estabilidad, la frecuencia con la que se suele realizar la prueba o examen y cualquier otro requisito importante, como la facilidad para recoger la muestra (p.ej. estudios en pacientes pediátricos) o (p.ej. pruebas genéticas, oncológicas e histopatológicas), que por otro lado suelen venir establecidas por ley [11].

### Ejemplo de tiempo de retención

En el Material Suplementario, así como en los Apéndices A y B, respectivamente, ser proponen tiempos de retención para los elementos documentales del sistema de calidad y para muestras y objetos de examen. Los tiempos de retención del análisis de riesgos que se muestra como ejemplo no deben considerarse una guía. En comparación con los resultados de la encuesta, en la mayoría de los casos, la evaluación de riesgos ha demostrado ser un método más ágil a la hora de establecer los tiempos de retención.

## Conclusiones

Los laboratorios clínicos documentan sus actividades para garantizar la calidad permanente del servicio, entre otras cosas. EFLM WG-A/ISO propone que los laboratorios clínicos adopten la metodología basada en el análisis de riesgos descrita en el presente documento, para definir unos planes de retención apropiados y específicos para la documentación, solicitudes, registros y muestras.

Los tiempos de retención establecidos en el Material Suplementario, así como en los Apéndices A y B, respectivamente, para los elementos documentales del sistema de calidad y para muestras y objetos de examen no deben considerarse una guía. Es esencial que cada laboratorio realice su propia evaluación de riesgos específica sobre uso clínico y analítico. La legislación vigente prevalecerá sobre nuestra propuesta relativa a la forma de establecer tiempos de retención adecuados. Resumir la legislación actual correspondiente se encontraba fuera del ámbito de este estudio (por ejemplo, en las pruebas genéticas o las muestras para los bancos de sangre).

## Supplementary Material

Supplementary Material Details

Supplementary Material Details

Supplementary Material Details

## Material Suplementario

Este artículo incluye material suplementario (https://doi.org/10.1515/almed-2024-0020).
