# Supplementary material for: Grupo de Trabajo de la EFLM sobre Acreditación y Normas ISO/CEN sobre cómo abordar los requisitos de la norma ISO15189 sobre retención de documentación y muestras
Source: Adv Lab Med. 2024 Mar 8;5(2):109–14. [Article in Spanish] doi: 10.1515/almed-2024-0020 (PMC11206178; doi:10.1515/almed-2024-0020)
Supplement: Supplementary file 2 — Supplementary Material Details [file j_almed-2024-0020_suppl_002.doc]

Apéndice A:

Tiempos de retención de muestras para elementos del sistema de calidad documental

Según la norma ISO 15189 se deberían retener los siguientes documentos de gestión general de la calidad. El laboratorio clínico deberá definir sus propios tiempos de retención para dichos documentos, excepto en caso de existir requerimiento legal.

Documentos de gestión general de la calidad:

Como ejemplo, presentamos los periodos de retención (en años, salvo que se especifique lo contrario):

- Entidad jurídica - al menos cinco años

- Manual de calidad – cinco años

- Procedimientos - cinco años

- Documentación para la planificación, funcionamiento y control de procesos analíticos cinco años

- Copias de reglamentos, normas y otros documentos normativos aplicables – cinco años

Registros:

Los periodos de retención se presentan a modo de ejemplo (en años, salvo que se especifique lo contrario):

- Selección y eficacia del proveedor y cambios en la lista aprobada de proveedores – cinco años

- Registros sobre cualificaciones, formación y competencia (registros de personal)-empleo más de un año

- Peticiones de examen- ocho semanas

- Registro de recepción de muestras en el laboratorio - ocho semanas

- Información sobre los reactivos y materiales empleados para la prueba (p.ej. documentación sobre el lote, certificados de suministros, prospectos, etc.) – cinco años

- Cuadernos u hojas de trabajo de laboratorio – un año

- Impresiones de instrumentos y datos e información retenidos - un año

- Resultados e informes de la prueba – cinco años

- Registros de mantenimiento de instrumentos, incluyendo registros de calibración interna y externa - durante la vida útil del equipo más un año

- Funciones de calibración y factores de conversión – cinco años

- Registros de control interno de calidad - un año

- Registros de incidencias y acciones emprendidas – cinco años

- Registros de accidentes y acciones emprendidas – cinco años

- Registros de gestión de riesgos – cinco años

- Diconformidades identificadas y acciones inmediatas emprendidas – cinco años

- Acciones preventivas emprendidas – cinco años

- Reclamaciones y acciones emprendidas – cinco años

- Registros de auditorías internas y externas – cinco años

- Comparaciones entre laboratorios de resultados de pruebas – cinco años

- Registros de actividades de mejora de la calidad – cinco años

- Actas de reuniones que registran decisiones adoptadas en relación a actividades de gestión de calidad del laboratorio – cinco años

- Registros de revisiones de gestión – cinco años

- Informes - (como parte de la documentación del paciente, dependiendo de la legislación nacional )
