# Supplementary material for: Grupo de Trabajo de la EFLM sobre Acreditación y Normas ISO/CEN sobre cómo abordar los requisitos de la norma ISO15189 sobre retención de documentación y muestras
Source: Adv Lab Med. 2024 Mar 8;5(2):109–14. [Article in Spanish] doi: 10.1515/almed-2024-0020 (PMC11206178; doi:10.1515/almed-2024-0020)
Supplement: Supplementary file 3 — Supplementary Material Details [file j_almed-2024-0020_suppl_003.doc]

Apéndice B:

Tiempos de retención para las muestras clínicas

El laboratorio clínico deberá definir sus propios tiempos de retención para las muestras clínicas, excepto si existe requerimiento legal. Los tiempos de retención dependen de la naturaleza de la muestra, su estabilidad, la frecuencia con la que se suele realizar la prueba o examen y cualquier otro requisito importante o reglamento especial aplicable (p.ej. estudios genéticos, estudios en pacientes pediátricos).

- General (incluyendo química clínica e inmunología): siete días desde la fecha de recepción (o hasta transcurridos dos días desde la fecha del informe, en caso de que este periodo fuera mayor). Las muestras se deberán almacenar en condiciones adecuadas, permitiendo su recuperación en condiciones de fiabilidad (se suele permitir descartar las muestras de orina enviadas para las pruebas con tira reactiva, el plasma para las pruebas rutinarias de coagulación, la sangre citratada para la EEB y el líquido cefalorraquídeo para la cuantificación de células mucho antes)

- Hematología: Muestras de sangre: siete días (para fines de identificación y trazabilidad); muestras de plasma para las pruebas de hemostasia: siete días (para fines de identificación y trazabilidad); pruebas especiales: congeladas: un mes; frotis de sangre: un mes; portaobjetos de médula ósea: 3 años.
